# Supplementary material for: Comprehensive Influences of Overexpression of a MYB Transcriptor Regulating Anthocyanin Biosynthesis on Transcriptome and Metabolome of Tobacco Leaves
Source: Int J Mol Sci. 2019 Oct 16;20(20):5123. doi: 10.3390/ijms20205123 (PMC6829574; doi:10.3390/ijms20205123)
Supplement: Supplementary file 1 [file ijms-20-05123-s001.zip › supplement files/Fig. S1. The TIC overlaps by QC sample Mass Spectrometry.docx]

A


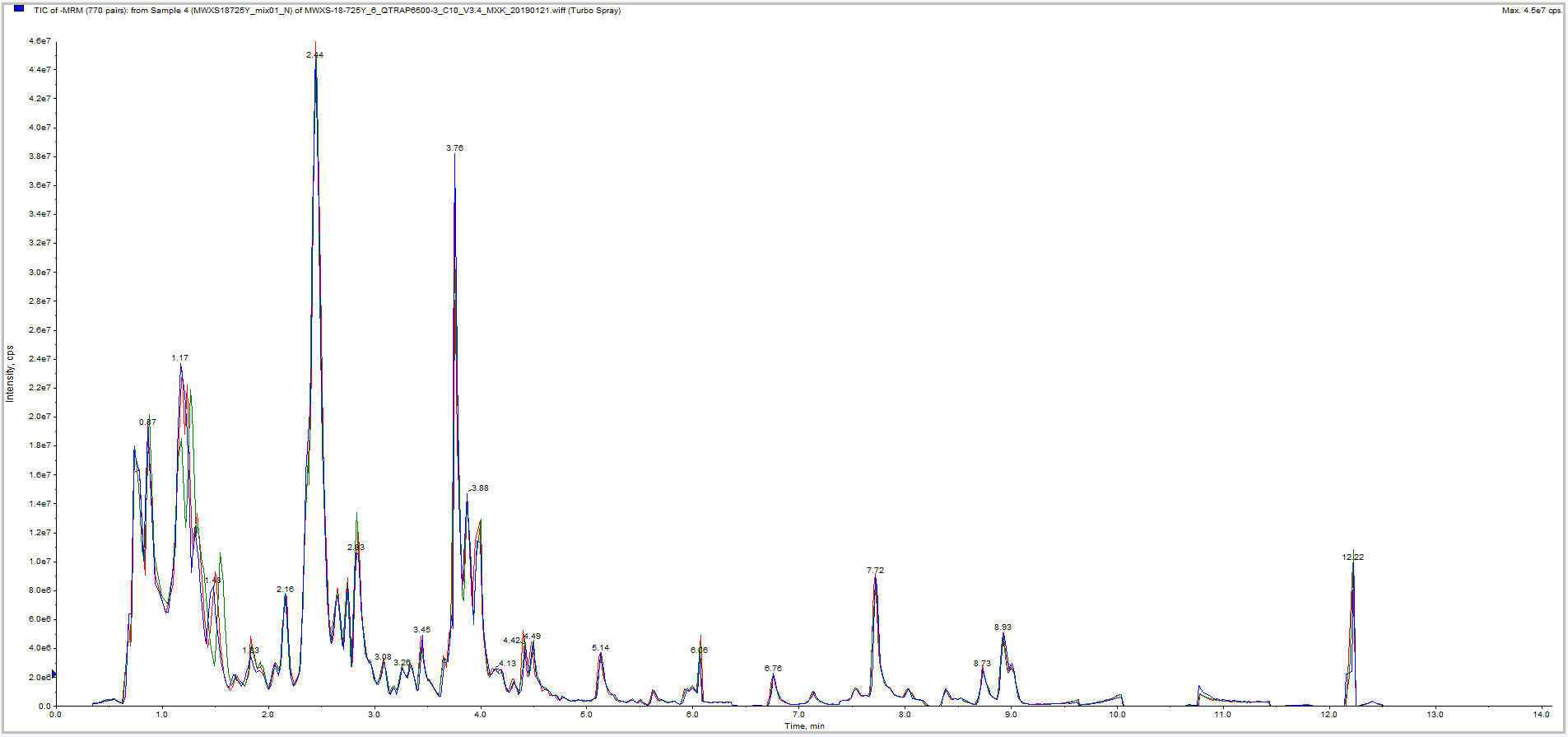


B


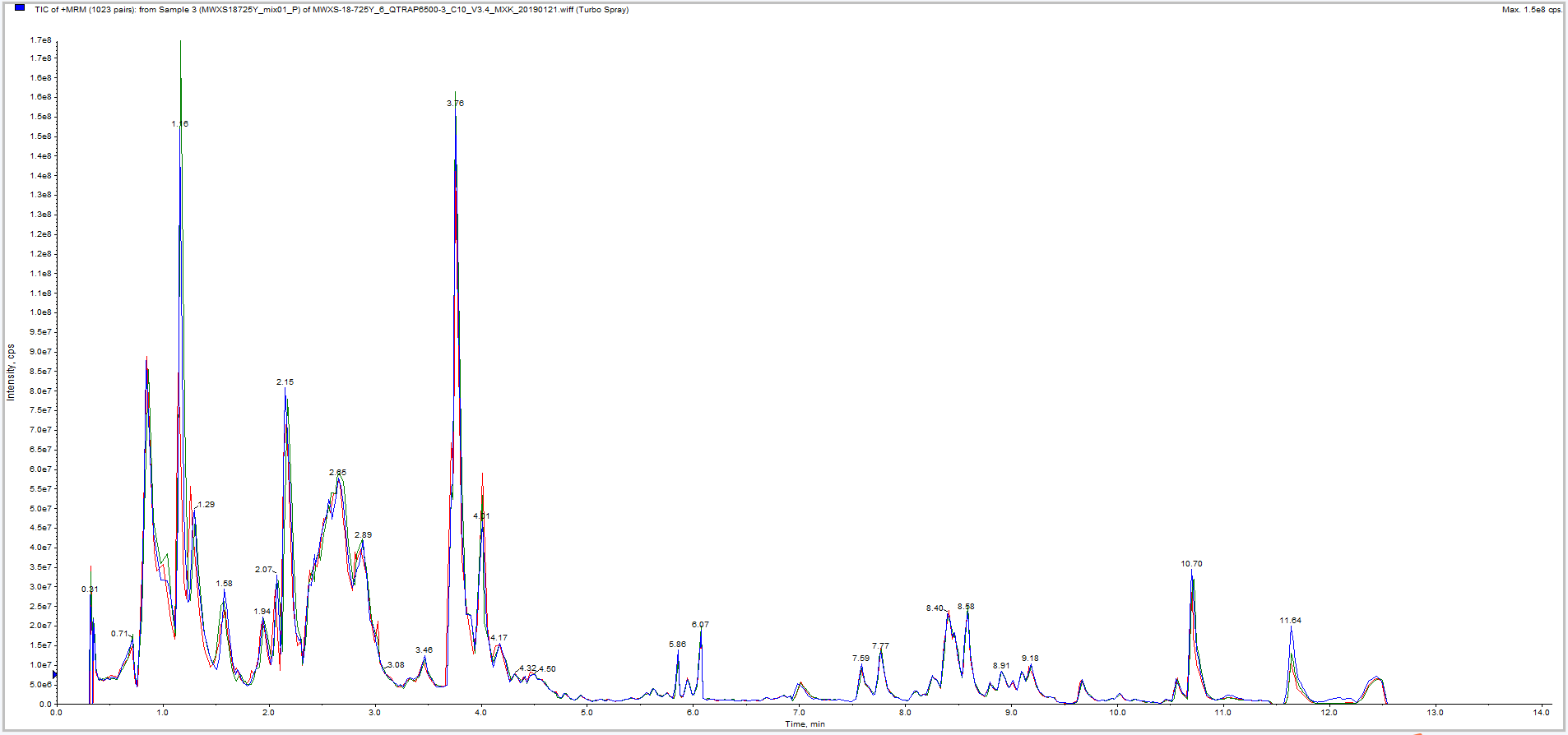


Fig. S1. The TIC overlaps by QC sample Mass Spectrometry. The pictures of (A) Transgenic lines (B) WT shows that the curves of total ion current detected by metabolite were highly overlapping. The retention time and peak intensity were consistent, indicating that the signal stability number of the same sample is detected by mass spectrometry at different time. The high stability of the instrument provides an important guarantee for the repeatability and reliability of the data.
