# Supplementary material for: Comprehensive Influences of Overexpression of a MYB Transcriptor Regulating Anthocyanin Biosynthesis on Transcriptome and Metabolome of Tobacco Leaves
Source: Int J Mol Sci. 2019 Oct 16;20(20):5123. doi: 10.3390/ijms20205123 (PMC6829574; doi:10.3390/ijms20205123)
Supplement: Supplementary file 1 [file ijms-20-05123-s001.zip › supplement files/Table S1. The primers used in this manuscript.docx]

Table S1. The primers used in this manuscript

| Gene | Primers | |
| --- | --- | --- |
|  | Forward | Reverse |
| Actin | AATGATCGGAATGGAAGCTG | TGGTACCACCACTGAGGACA |
| CHI | GAAATCCTCCGATCCAGTGA | CAACGTTGACAACATCAGGC |
| CHS | AGGAAAAGCCTTGTGGAAGCA | ACTTGGTCCAAAATTGCAGG |
| F3H | ACAGGGTGAAGTGGTCCAAG | CCTTGGTTAAGGCCTCCTTC |
| F3'H | TCCAAGAATACTGGCCCAAG | CTCACAACTCTCGGATGCAA |
| F3'5'H | GCCATAGATACAAGCCTCTT | ACCTAGAAGAGGAAGAGCGC |
| DFR | TCCCATCATGCGATCATCTA | ATGGCTTCTTTGTCACGTCC |
| ANS | TGGCGTTCAAGCTCATACTG | TTTACCGGGTGTCCCCAATA |
| AN2 | CTATTACTAAATCATCTGGAGTGAG | TTGATGCCATTTTCCTTCAC |
| AN1b | CTAGGGATTATCTGATGTATTG | TCTTCTTGGCTGAGTTAAGA |
| MYB3 | ATGAGAAAAACTTGCTGCGA | CCAGCAACCTTCTCCATGTT |
| AN2cds | TGTTCTTAATGCTACTGATGG | ATGATGAATACTAGTGTTACTAT |
| AN2attb | AAAAAGCAGGCTTCATGATGAATACTAGTGTTAC | AGAAAGCTGGGTCCTAATTCAGTAGATTCCATA |
| Attb adapter | GGGGACAAGTTTGTACAAAAAAGCAGGCT | GGGGACCACTTTGTACAAGAAAGCTGGGT |
