# Supplementary material for: Comprehensive Influences of Overexpression of a MYB Transcriptor Regulating Anthocyanin Biosynthesis on Transcriptome and Metabolome of Tobacco Leaves
Source: Int J Mol Sci. 2019 Oct 16;20(20):5123. doi: 10.3390/ijms20205123 (PMC6829574; doi:10.3390/ijms20205123)
Supplement: Supplementary file 1 [file ijms-20-05123-s001.zip › supplement files/Table S3.docx]

Table S3. Summary of transcriptome sequencing data in the Purple and Green.

| sample | total raw reads (Mb) | total clean reads (Mb) | total clean bases (Gb) | clean reads Q20(%) | clean reads Q30(%) | clean reads ratio (%) |
| --- | --- | --- | --- | --- | --- | --- |
| LrAN2-1 | 44.01 | 44 | 6.6 | 98.19 | 94.83 | 100 |
| LrAN2-2 | 42.28 | 42.28 | 6.34 | 98.03 | 94.41 | 100 |
| LrAN2-3 | 43.62 | 43.62 | 6.54 | 98.01 | 94.35 | 100 |
| WT-1 | 44.86 | 44.85 | 6.73 | 97.69 | 93.38 | 99.99 |
| WT-2 | 41.26 | 41.26 | 6.19 | 97.57 | 93.11 | 99.99 |
| WT-3 | 43.24 | 43.23 | 6.49 | 98.08 | 94.55 | 100 |
