# Supplementary material for: Comprehensive Influences of Overexpression of a MYB Transcriptor Regulating Anthocyanin Biosynthesis on Transcriptome and Metabolome of Tobacco Leaves
Source: Int J Mol Sci. 2019 Oct 16;20(20):5123. doi: 10.3390/ijms20205123 (PMC6829574; doi:10.3390/ijms20205123)
Supplement: Supplementary file 1 [file ijms-20-05123-s001.zip › supplement files/Table S4.docx]

Table S4. Distribution of the lengths of the assembled unigenes

| sample | total number | total length | mean length | N50 | N70 | N90 | GC(%) |
| --- | --- | --- | --- | --- | --- | --- | --- |
| LrAN2-1 | 93345 | 89155229 | 955 | 1594 | 975 | 380 | 39.95 |
| LrAN2-2 | 91820 | 85827923 | 934 | 1553 | 947 | 372 | 40.03 |
| LrAN2-3 | 96358 | 94725949 | 983 | 1663 | 1014 | 391 | 39.89 |
| WT-1 | 95557 | 91780595 | 960 | 1617 | 979 | 381 | 39.94 |
| WT-2 | 92186 | 87665381 | 950 | 1585 | 978 | 377 | 40.11 |
| WT-3 | 96485 | 94295406 | 977 | 1630 | 1009 | 392 | 39.9 |
| All-Unigene | 160965 | 198604906 | 1233 | 2002 | 1352 | 561 | 39.65 |
