# Supplementary material for: Comprehensive Influences of Overexpression of a MYB Transcriptor Regulating Anthocyanin Biosynthesis on Transcriptome and Metabolome of Tobacco Leaves
Source: Int J Mol Sci. 2019 Oct 16;20(20):5123. doi: 10.3390/ijms20205123 (PMC6829574; doi:10.3390/ijms20205123)
Supplement: Supplementary file 1 [file ijms-20-05123-s001.zip › supplement files/Table S5. The annotation of assembled unigenes.docx]

Table S5. The annotation of assembled unigenes

| values | total | Nr | Nt | Swissprot | KEGG | KOG | Interpro | GO | Inter | Overall |
| --- | --- | --- | --- | --- | --- | --- | --- | --- | --- | --- |
| Number | 160,965 | 103,854 | 132,624 | 64,799 | 73,083 | 69,940 | 61,708 | 67,940 | 35,629 | 135,911 |
| Percentage | 100% | 64.52% | 82.39% | 40.26% | 45.40% | 43.45% | 38.34% | 42.21% | 22.13% | 84.44% |
