# Supplementary material for: Comprehensive Influences of Overexpression of a MYB Transcriptor Regulating Anthocyanin Biosynthesis on Transcriptome and Metabolome of Tobacco Leaves
Source: Int J Mol Sci. 2019 Oct 16;20(20):5123. doi: 10.3390/ijms20205123 (PMC6829574; doi:10.3390/ijms20205123)
Supplement: Supplementary file 1 [file ijms-20-05123-s001.zip › supplement files/Table S7.docx]

Table S7. The relative transcript level of the unigenes relative to anthocyanin biosynthesis

| Gene | Gene_id | FPKM value of transgenic lines | FPKM value of WT | FoldChange | log2FoldChange |
| --- | --- | --- | --- | --- | --- |
| PAL | CL7840.Contig12_All | 35.22 | 29.18 | 1.71 | 0.27 |
|  | CL7840.Contig9_All | 26.46 | 23.28 |  | 0.18 |
|  | CL7840.Contig7_All | 15.91 | 8.51 |  | 0.9 |
|  | CL7840.Contig10_All | 27.61 | 28.75 |  | -0.06 |
|  | CL7840.Contig8_All | 8.38 | 6.91 |  | 0.28 |
|  | CL7840.Contig5_All | 3.06 | 2.84 |  | 0.11 |
|  | CL7840.Contig11_All | 29.68 | 7.25 |  | 2.03 |
|  | CL7840.Contig2_All | 20.97 | 18.51 |  | 0.18 |
|  | CL7840.Contig3_All | 8.75 | 8.46 |  | 0.05 |
|  | CL7840.Contig4_All | 64.44 | 5.23 |  | 3.62 |
|  | CL7840.Contig6_All | 1.83 | 2.39 |  | -0.39 |
| C4H | CL10654.Contig3_All | 99.91 | 43.31 | 2.03 | 1.21 |
|  | Unigene15500_All | 6.60 | 9.15 |  | -0.47 |
| 4CL | Unigene40595_All | 10.47 | 14.90 | 0.72 | -0.51 |
|  | Unigene40597_All | 3.95 | 5.22 |  | -0.40 |
| CHS | Unigene26075_All | 195.53 | 46.22 | 5.16 | 2.08 |
|  | Unigene26078_All | 289.53 | 28.92 |  | 3.32 |
|  | Unigene26079_All | 302.73 | 83.65 |  | 1.85 |
|  | Unigene26077_All | 244.21 | 22.36 |  | 3.45 |
| CHI | CL2294.Contig2_All | 71.74 | 58.52 | 1.21 | 0.29 |
|  | CL2294.Contig3_All | 29.11 | 24.81 |  | 0.23 |
| F3H | CL18244.Contig2_All | 81.53 | 82.93 | 0.98 | -0.02 |
| F3'H | CL14621.Contig4_All | 93.5 | 18.37 | 4.62 | 2.35 |
|  | CL14621.Contig2_All | 94.22 | 14.87 |  | 2.66 |
|  | CL14621.Contig3_All | 4.71 | 6.56 |  | -0.48 |
| F3'5'H | Unigene31620_All | 2.64 | 2.94 | 0.90 | -0.15 |
| DFR | CL4211.Contig4_All | 60.93 | 7.37 | 330.26 | 3.05 |
|  | CL4211.Contig1_All | 1305.57 | 8.65 |  | 7.24 |
|  | CL4211.Contig3_All | 42.10 | 7.97 |  | 2.40 |
|  | CL4211.Contig2_All | 7273.08 | 14.18 |  | 9.00 |
| ANS | CL13975.Contig3_All | 23196.78 | 37.89 | 471.46 | 9.25 |
|  | CL13975.Contig1_All | 659.09 | 12.71 |  | 5.70 |
|  | CL17146.Contig3_All | 1.85 | 8.81 |  | -2.25 |
| AN2 | Unigene39134_All | 1849.43 | 9.13 | 202.57 | 7.67 |
| MYB3 | Unigene21182_All | 16.37 | 3.35 | 23.82 | 2.29 |
|  | CL4559.Contig4_All | 25.39 | 5.53 |  | 2.20 |
|  | CL4559.Contig2_All | 466.30 | 24.23 |  | 4.27 |
|  | CL4559.Contig3_All | 278.14 | 11.58 |  | 4.59 |
|  | CL4559.Contig7_All | 736.17 | 17.78 |  | 5.37 |
|  | Unigene56315_All | 729.93 | 21.26 |  | 5.10 |
|  | CL4559.Contig6_All | 130.94 | 13.00 |  | 3.33 |
|  | CL4559.Contig1_All | 43.90 | 4.02 |  | 3.45 |
|  | CL4559.Contig5_All | 441.77 | 17.04 |  | 4.70 |
|  | Unigene21182_All | 16.37 | 3.35 |  | 2.29 |
| CPC | CL16341.Contig2_All | 18.38 | 3.43 | 5.35 | 2.42 |
| AN1b | CL20065.Contig3_All | 415.35 | 15.84 | 36.88 | 4.71 |
|  | CL20065.Contig8_All | 884.08 | 20.98 |  | 5.40 |
|  | CL20065.Contig5_All | 163.89 | 8.39 |  | 4.29 |
|  | CL20065.Contig6_All | 236.94 | 11.85 |  | 4.32 |
|  | CL20065.Contig4_All | 309.96 | 41.69 |  | 2.89 |
|  | CL20065.Contig7_All | 1074.47 | 9.67 |  | 6.80 |
|  | CL20065.Contig10_All | 582.60 | 11.08 |  | 5.72 |
|  | CL20065.Contig1_All | 648.98 | 7.39 |  | 6.46 |
|  | CL20065.Contig9_All | 270.00 | 7.22 |  | 5.23 |
|  | CL20065.Contig2_All | 663.23 | 8.23 |  | 6.33 |
