# Supplementary material for: Comprehensive Influences of Overexpression of a MYB Transcriptor Regulating Anthocyanin Biosynthesis on Transcriptome and Metabolome of Tobacco Leaves
Source: Int J Mol Sci. 2019 Oct 16;20(20):5123. doi: 10.3390/ijms20205123 (PMC6829574; doi:10.3390/ijms20205123)
Supplement: Supplementary file 1 [file ijms-20-05123-s001.zip › supplement files/Table S8.docx]

Table S8. The KEGG pathways of differently expression unigenes and differently accumulation chemical compounds

| Kegg pathway | Ko id | P-value  gene | P-value  meta | gene ID | gene Count | gene Annotation | meta ID | meta Count | meta Annotation |
| --- | --- | --- | --- | --- | --- | --- | --- | --- | --- |
| Fatty acid degradation | ko00071 | 0.10 | 0.67 | Unigene33722_All; Unigene33721_All | 2 | K01897, K01897 | pme0243 | 1 | Glutaric acid |
| Glycine, serine and threonine metabolism | ko00260 | 0.60 | 0.46 | CL12399.Contig4_All | 1 | K19996 | pme1828; pme0050; pme0011; pme1851 | 4 | Betaine, L-Tryptophan, L-Aspartic acid, Creatine |
| Monobactam biosynthesis | ko00261 | 0.14 | 0.89 | CL12399.Contig4_All | 1 | K19996 | pme0011 | 1 | L-Aspartic acid |
| Cysteine and methionine metabolism | ko00270 | 0.39 | 0.99 | CL12399.Contig4_All; Unigene15411_All | 2 | K19996, K01011 | pme0011 | 1 | L-Aspartic acid |
| Lysine biosynthesis | ko00300 | 0.01 | 0.89 | CL12399.Contig4_All; CL7632.Contig5_All | 2 | K19996, K10206 | pme0011 | 1 | L-Aspartic acid |
| Phenylalanine metabolism | ko00360 | 0.35 | 0.74 | CL7840.Contig4_All; CL7840.Contig11_All | 2 | K10775, K10775 | pme2903; pme0177; pme2366; pme1436 | 4 | 4-Hydroxybenzoic acid, Phenylacetyl-L-glutamine, Phenethylamine, p-Coumaric acid |
| Cyanoamino acid metabolism | ko00460 | 0.00 | 0.95 | Unigene10476_All; CL721.Contig8_All; CL721.Contig10_All; CL721.Contig6_All; CL721.Contig7_All; CL721.Contig3_All | 6 | K12153, K01188, K01188, K01188, K01188, K01188 | pme0011 | 1 | L-Aspartic acid |
| Amino sugar and nucleotide sugar metabolism | ko00520 | 0.31 | 0.93 | Unigene19543_All; CL16572.Contig1_All; Unigene16808_All; CL1626.Contig7_All | 4 | K01183, K01183, K16613, K07305 | pmb0786 | 1 | Glucosamine |
| Phenylpropanoid biosynthesis | ko00940 | 0.00 | 0.20 | CL7840.Contig4_All; CL7840.Contig11_All; CL721.Contig8_All; CL721.Contig10_All; CL721.Contig6_All; CL721.Contig7_All; CL721.Contig3_All | 7 | K10775, K10775, K01188, K01188, K01188, K01188, K01188 | pme0305; pma0149; pme1436; pmf0285; pme2213; pme2993; pmf0014 | 7 | Ferulic acid, Sinapoyl malate, p-Coumaric acid, 4-Hydroxy-3-methoxycinnamaldehyde, Caffeate, Scopoletin (7-Hydroxy-5-methoxycoumarin), Scopolin |
| Flavonoid biosynthesis | ko00941 | 0.00 | 0.00 | CL4211.Contig4_All; CL4211.Contig1_All; CL14621.Contig1_All; CL13975.Contig3_All; CL13975.Contig1_All; Unigene34084_All; CL4211.Contig3_All; CL4211.Contig2_All | 8 | K13082, K13082, K05280, K05277, K05277, K00660, K13082, K13082 | pmf0345; pme0088; pmf0583; pme1201; pme0201; pme1535; pme3396; pme3475; pme2960; pme1521; pme1399; pme1514; pme0377; pme0450; pme0330; pme0442; pme2895; pme1478; pme0372; pme2963; pme0199 | 21 | (-)-Epiafzelechin, Luteolin, Phloridzin, Phloretin, Catechin, (, )-Gallocatechin (GC), Fustin, Butin, Naringenin chalcone, Dihydroquercetin (Taxifolin), Xanthohumol, Epigallocatechin (EGC), Naringenin, L-Epicatechin, Naringenin 7-O-neohesperidoside (Naringin), Delphinidin, Dihydromyricetin, Myricetin, Naringenin 7-O-glucoside (Prunin), Aromadedrin (Dihydrokaempferol), Quercetin |
| Flavone and flavonol biosynthesis | ko00944 | 0.04 | 0.31 | CL14621.Contig1_All | 1 | K05280 | pme0199; pme1478; pmf0471; pme3297; pmb0605; pme0088 | 6 | Quercetin, Myricetin, Apiin, Kaempferol 3-O-rhamnoside (Kaempferin), Apigenin 7-O-glucoside (Cosmosiin), Luteolin |
| Glucosinolate biosynthesis | ko00966 | 0.33 | 0.89 | Unigene10476_All | 1 | K12153 | pme0050 | 1 | L-Tryptophan |
| Aminoacyl-tRNA biosynthesis | ko00970 | 0.69 | 0.97 | CL12399.Contig4_All | 1 | K19996 | pme0011; pme0050; pme0006 | 3 | L-Aspartic acid, L-Tryptophan, L-Proline |
| Metabolic pathways | ko01100 | 0.19 | 0.99 | CL4211.Contig4_All; CL12399.Contig4_All; CL7840.Contig4_All; Unigene38174_All; Unigene19543_All; CL19329.Contig5_All; Unigene4386_All; CL20065.Contig7_All; CL20065.Contig10_All; CL7840.Contig11_All; CL721.Contig8_All; CL4211.Contig1_All; Unigene15411_All; CL7632.Contig5_All; CL721.Contig10_All; Unigene33722_All; CL14621.Contig1_All; CL20065.Contig2_All; CL20065.Contig9_All; CL13975.Contig3_All; CL721.Contig6_All; CL13975.Contig1_All; CL721.Contig7_All; CL16572.Contig1_All; Unigene33721_All; Unigene16808_All; CL721.Contig3_All; Unigene34084_All; CL4211.Contig3_All; CL20065.Contig1_All; CL1626.Contig7_All; CL4211.Contig2_All; CL12809.Contig12_All | 33 | K13082, K19996, K10775, K01187, K01183, K01369, K01214, K13422, K13422, K10775, K01188, K13082, K01011, K10206, K01188, K01897, K05280, K13422, K13422, K05277, K01188, K05277, K01188, K01183, K01897, K16613, K01188, K00660, K13082, K13422, K07305, K13082, K01214 | pmb3101; pme1521; pme0245; pmb0829; pmf0285; pme0088; pme1436; pme1292; pme1306; pmb0247; pme0230; pme0199; pme1814; pme0282; pme1313; pme2765; pme1119; pmb0786; pme0006; pme1952; pme2960; pmf0213; pme0011; pme2486; pme0305; pme0234; pme1383; pme2213; pme2963; pme1851; pme0274; pme1055; pme2607; pme1828; pme3184; pme2746; pme2266; pme2903; pmf0604; pme0050; pme0377; pmf0214 | 42 | 2-Isopropylmalate, Dihydroquercetin (Taxifolin), Adipic acid, Phosphoric acid, 4-Hydroxy-3-methoxycinnamaldehyde, Luteolin, p-Coumaric acid, Homogentisic acid, Pyridoxine 5'-phosphate, p-Aminobenzoate, Adenosine, Quercetin, 2,5-dihydroxybenzoic acid (Gentisic acid), Phthalic acid, N'-Formylkynurenine, 1,7-Dimethylxanthine, Inosine, Glucosamine, L-Proline, Riboflavin, Naringenin chalcone, Caffeine, L-Aspartic acid, Protocatechuic acid, Ferulic acid, Kynurenic acid, Pyridoxine, Caffeate, Aromadedrin (Dihydrokaempferol), Creatine, 6-Aminocaproic acid, L-Kynurenine, 5-Hydroxyindole-3-acetic acid, Betaine, 2'-Deoxyadenosine-5'-monophosphate, Flavin adenine dinucleotide (FAD), Biotin, 4-Hydroxybenzoic acid, β-Caryophyllene, L-Tryptophan, Naringenin, Theophylline |
| Biosynthesis of secondary metabolites | ko01110 | 0.00 | 0.49 | CL4211.Contig4_All; Unigene10476_All; CL12399.Contig4_All; CL7840.Contig4_All; CL19329.Contig5_All; Unigene4386_All; CL20065.Contig7_All; CL20065.Contig10_All; CL7840.Contig11_All; CL721.Contig8_All; CL4211.Contig1_All; CL7632.Contig5_All; CL721.Contig10_All; CL14621.Contig1_All; CL20065.Contig2_All; CL20065.Contig9_All; CL13975.Contig3_All; CL721.Contig6_All; CL13975.Contig1_All; CL721.Contig7_All; CL721.Contig3_All; Unigene34084_All; CL4211.Contig3_All; CL20065.Contig1_All; CL4211.Contig2_All; CL12809.Contig12_All | 26 | K13082, K12153, K19996, K10775, K01369, K01214, K13422, K13422, K10775, K01188, K13082, K10206, K01188, K05280, K13422, K13422, K05277, K01188, K05277, K01188, K01188, K00660, K13082, K13422, K13082, K01214 | pme0199; pmf0014; pme0006; pme1514; pme2765; pme1521; pmb3101; pme2706; pme0088; pme1436; pmf0285; pme0201; pme2746; pme1478; pme2213; pme2963; pmf0214; pme0442; pme0050; pme0450; pme0377; pme2903; pmf0604; pme2895; pmf0213; pme2960; pme1952; pme2993; pme1201; pme0305; pmf0345; pmf0583; pme2486; pme0011; pme1535 | 35 | Quercetin, Scopolin, L-Proline, Epigallocatechin (EGC), 1,7-Dimethylxanthine, Dihydroquercetin (Taxifolin), 2-Isopropylmalate, 2,3-Dihydroxybenzoic acid, Luteolin, p-Coumaric acid, 4-Hydroxy-3-methoxycinnamaldehyde, Catechin, Flavin adenine dinucleotide (FAD), Myricetin, Caffeate, Aromadedrin (Dihydrokaempferol), Theophylline, Delphinidin, L-Tryptophan, L-Epicatechin, Naringenin, 4-Hydroxybenzoic acid, β-Caryophyllene, Dihydromyricetin, Caffeine, Naringenin chalcone, Riboflavin, Scopoletin (7-Hydroxy-5-methoxycoumarin), Phloretin, Ferulic acid, (-)-Epiafzelechin, Phloridzin, Protocatechuic acid, L-Aspartic acid, (, )-Gallocatechin (GC) |
| 2-Oxocarboxylic acid metabolism | ko01210 | 0.30 | 0.92 | Unigene10476_All; CL12399.Contig4_All | 2 | K12153, K19996 | pmb3101; pme0011; pme0050 | 3 | 2-Isopropylmalate, L-Aspartic acid, L-Tryptophan |
| Biosynthesis of amino acids | ko01230 | 0.83 | 1.00 | CL12399.Contig4_All; CL7632.Contig5_All | 2 | K19996, K10206 | pme0006; pme0050; pmb3101; pme0011 | 4 | L-Proline, L-Tryptophan, 2-Isopropylmalate, L-Aspartic acid |
